# *Announcement*: World Birth Defects Day — March 3, 2017

**DOI:** 10.15585/mmwr6607a4

**Published:** 2017-02-24

Every year, approximately 3%–6% of infants worldwide are born with a serious birth defect (*1*–*5*). Birth defects can affect an infant regardless of birthplace, race, or ethnicity. In many countries, birth defects are among the leading causes of death for infants and young children (*6*). Those who survive and live with these conditions are at an increased risk for lifelong disabilities.

During the past year, birth defects have received increased attention, as researchers at CDC and worldwide have been studying the relationship between Zika virus disease and congenital Zika syndrome (*7*). The Zika virus disease outbreak and its effect on birth defects have highlighted the need for and benefits of international collaboration and communication about birth defects prevention.

To further raise awareness about birth defects, 33 countries joined to support World Birth Defects Day in 2016 (*8*). On March 3, 2016, social media presence of the hashtag #WorldBDDay reached nearly 4.8 million persons around the world.

For World Birth Defects Day 2017, the same group of partners has reconvened and invited others to join them to continue to bring attention to this global public health issue. The goals for 2017 are to raise awareness about birth defects, reduce stigma, and increase opportunities for prevention by 1) increasing the number of birth defects surveillance programs globally, 2) improving existing birth defects surveillance programs, 3) improving access to care, and 4) continuing research on the causes of birth defects.

CDC invites other organizations around the world to participate in World Birth Defects Day 2017 by sharing stories and information about birth defects using the hashtag #WorldBDDay.
